# Structure Prediction of Partial-Length Protein Sequences

**DOI:** 10.3390/ijms140714892

**Published:** 2013-07-17

**Authors:** Adrian Laurenzi, Ling-Hong Hung, Ram Samudrala

**Affiliations:** 1 Department of Computer Science & Engineering, University of Washington, Box 352350, Seattle, WA 98195-2350, USA; E-Mail: alaurenz@uw.edu; 2 Department of Microbiology, University of Washington, Box 357242, Seattle, WA 98195-7242, USA; E-Mail: lhhunghimself@gmail.com

**Keywords:** protein structure prediction, EST, expressed sequence tag, protein folding, protein design

## Abstract

Protein structure information is essential to understand protein function. Computational methods to accurately predict protein structure from the sequence have primarily been evaluated on protein sequences representing full-length native proteins. Here, we demonstrate that top-performing structure prediction methods can accurately predict the partial structures of proteins encoded by sequences that contain approximately 50% or more of the full-length protein sequence. We hypothesize that structure prediction may be useful for predicting functions of proteins whose corresponding genes are mapped expressed sequence tags (ESTs) that encode partial-length amino acid sequences. Additionally, we identify a confidence score representing the quality of a predicted structure as a useful means of predicting the likelihood that an arbitrary polypeptide sequence represents a portion of a foldable protein sequence (“foldability”). This work has ramifications for the prediction of protein structure with limited or noisy sequence information, as well as genome annotation.

## 1. Introduction

Elucidation of protein structure is powerful for understanding the functions of biological macromolecules [1]. When experimentally determined structures are not available, computational methods can be used to accurately predict protein structures from the sequence [2]. Evaluation of structure prediction accuracy, such as that done every two years during the Critical Assessment of Techniques for Protein Structure Prediction (CASP) experiments, has only been conducted using full-length sequences or complete domains of known proteins (native sequences) [3]. We hypothesize that a sequence representing a sizable portion of a native sequence (subsequence) would tend to fold into a stable structure similar to the corresponding partial structure of the native protein. To investigate this, we evaluated the quality of structures predicted by top-performing protein structure prediction software given partial-length sequences taken from sequences of known proteins. Our results demonstrate that three top-performing structure prediction methods, I-TASSER [4], Rosetta [5] and ProtinfoCM [6], combined with HHpred [7] (ProtinfoCM/HH), can predict partial structures of proteins encoded by sequences that contain approximately 50% or more of the native sequence with comparable accuracy to predictions made from native sequences. This finding led us to develop two novel applications of the protein structure prediction software described in the following sections.

### 1.1. Predicting the Foldability of Polypeptide Sequences

Predicting the dynamics of protein folding is essential for understanding biology at the cellular level. Genes have evolved to encode specific sequences of amino acids that fold into stable macromolecular structures. Although functional proteins have been identified that are intrinsically disordered, which lack a stable tertiary structure in their native state [8], functional proteins typically have a relatively stable structure. Given an arbitrary polypeptide sequence, it would be useful to accurately predict the likelihood that the sequence represents a portion of a foldable protein sequence. This knowledge would be potentially useful in genome annotation by supporting or rejecting hypothetical proteins derived from unknown regions of DNA.

FoldIndex [9] is an existing method that predicts if a given protein sequence assumes a defined fold (”foldability”) based on the net charge and average hydrophobicity of the amino acids. The method was effective in differentiating between protein sequences known to be intrinsically disordered and those that are not. Methods also exist that predict disordered regions of proteins by assigning a disorder score to each residue, but they do not give a single foldability score for the protein [10].

To our knowledge, FoldIndex is the only method that outputs a single score representing the foldability of a given sequence. Because FoldIndex was evaluated exclusively on protein sequences found in nature, it may not reliably predict the foldability of non-native sequences. It would be useful in protein design to reliably predict if an arbitrary sequence, native or nonnative, would fold to produce a stable structure. We introduce an approach for predicting the foldability of an arbitrary polypeptide sequence by exploiting information from protein structure predictions. We demonstrate that C-score, a score that estimates the quality of a structure predicted by I-TASSER, may be useful in predicting if an arbitrary polypeptide sequence is foldable, because it can reliably discriminate between sequences taken from known proteins, native, as well as partial-length non-native sequences and sequences created by shuffling the residues of those sequences. We make the assumption that, in general, the shuffled sequences would not assume a defined fold and, thus, are not foldable. This assumption is reasonable based on a study [11] that investigated the folding behavior of 79 totally random polypeptide sequences, each 50 amino acids in length, and found that only 20% of the sequences were likely to have folded to some degree. Our results suggest that C-score could be used directly to rank a set of arbitrary sequences by the likelihood that they would fold to produce a stable tertiary structure.

### 1.2. Predicting Structures of Partial-Length Sequences to Improve EST Annotation

Despite the rapid increase in whole genome sequence data available, expressed sequence tag (EST) data remains an important and useful source of sequence information. Currently, there are over 70 million ESTs in GenBank representing nearly half of all GenBank entries [12]. While GenBank contains complete genomes for nearly 1600 different organisms, EST data exists for over 2200 organisms [13].

To make efficient use of EST data, computational strategies have been developed to analyze and organize EST data stored in large public databases [14–16]. EST data has been extremely useful in discovering new genes, understanding gene expression and regulation and constructing genome maps [17]. However, the utility of EST data relies upon the ability to make accurate annotations that describe the functionality of an EST in the source organism. The most common approach for predicting the function of an EST is to use a sequence-based comparison method, such as BLAST, to search ESTs against nucleotide or protein databases of known function [18]. However, when an EST sequence shows less than 30% sequence similarity to a protein with known function (the “twilight zone”), sequence-based annotation techniques are unlikely to be accurate [19]. In these cases, using protein structure prediction software to model the protein encoded by the EST (EST structure) could enable accurate annotation. An EST structure that is likely to be accurate, denoted for I-TASSER models by a high C-score, would rule out the possibility that the EST does not represent a real protein and also provide structure information to use for annotating the EST.

To predict EST structure, the protein-coding region of the EST must first be identified. Coding regions can be predicted using software, such as ORFpredictor [20] and ESTScan [21], which, given benchmarking datasets of assembled EST sequences, can predict coding regions with near-perfect accuracy. Since sequencing errors are common in ESTs, when assembled, high-quality EST data is not available; error tolerance in coding region prediction is important. Given low-quality ESTs, an error tolerant version of ESTScan can predict 64.2% of start sites and 55.1% of stop sites perfectly [22]. After the coding region is predicted, the amino acid sequence can be determined and input into protein structure prediction software, such as I-TASSER. Structure-based functional annotation methods could then be applied to the predicted structure to suggest possible functions of the EST.

It is well established that structure is more conserved than sequence [23,24], so having an accurately predicted structure of an uncharacterized protein-coding EST is likely to provide insight about function that the sequence alone could not afford. A myriad of publicly available computational methods have been developed to predict the function of a protein given its structure [1,25,26]. With the rapid accumulation of uncharacterized protein data [27], structure-based function prediction is a popular and active area of research. Structure-based methods that make predictions using templates of known function have been shown to outperform purely sequence-based methods [28,29]. Furthermore, one of the best-performing function prediction methods in the CASP9 experiment [3], a blind assessment of protein structure and function prediction, was COFACTOR [30], a structure-based method. Methods that predict functionally important residues have demonstrated that predictions made using sequence and structure information are more accurate than those made using only sequence information [31,32]. In addition to making accurate predictions, given structures that were determined experimentally, structure-based annotation methods perform with similar accuracy on predicted structures [29,33].

Unlike the protein sequences used to evaluate protein structure and function prediction methods, EST sequences often do not contain the entire coding region of the proteins they encode. Given our finding that structures can be reliably predicted from sequences containing approximately 50% or more of the native sequence, we suggest that structure prediction would be useful in annotating at least a subset of sequences from EST databases, especially those with unreliable annotations.

## 2. Results

### 2.1. Evaluation of Structure Prediction of Partial-Length Sequences

**Figure 1:**
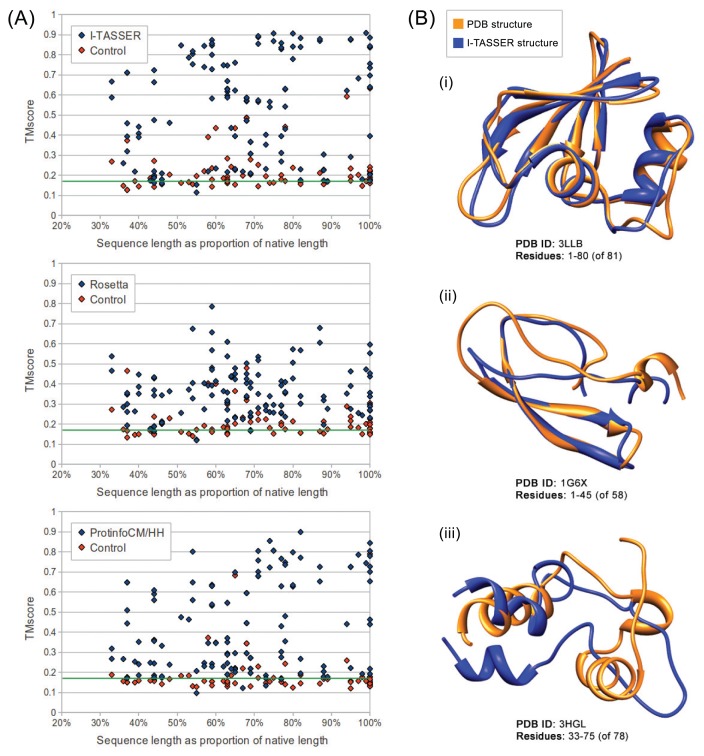
The performance of I-TASSER, Rosetta and ProtinfoCM/HH across all native sequences and subsequences. (**A**) The length of each subsequence, as a percentage of the length of the source native sequence it was taken from (one of the ten benchmarking Protein Data Bank (PDB) structures), is plotted against the TM-score calculated between the predicted partial structure and the corresponding partial structure from the native PDB structure. The TM-score is a number between zero and one representing the quality of the prediction. A TM-score around or below 0.17 (marked in green) means the prediction is no more accurate than making a random selection from the PDB. The points at the rightmost side of the plot (100%) represent the native sequences. Sequences created by shuffling the residues of each test sequence were used to generate control structures (indicated in red); (**B**) The best (i); average (ii) and worst (iii) subsequence predictions generated by I-TASSER (blue), the best-performing method, aligned, using UCSF Chimera [34], to the corresponding native PDB structures (orange). Overall, the structure of subsequences representing 50% or more of the full-length native sequence can be robustly predicted by all three structure prediction methods.

We evaluated the quality of structures predicted from sequences in our benchmarking dataset containing the sequences from ten maximally diverse structures from the PDB, 111 subsequences and 72 control sequences. Each subsequence was generated by slicing out a random region of contiguous sequences from one of the native sequences. Control sequences were generated by shuffling the native sequences and subsequences. The quality of structures predicted from each sequence in the dataset was evaluated using TM-score and MaxSub. Both are automated methods that assign a score between zero and one to a predicted model based on its similarity to the native structure. I-TASSER predictions generated from subsequences representing 50% or more of their native sequence were of comparable quality to predictions generated from native sequences with average TM-scores of 0.57 and 0.62, respectively. Rosetta had poorer performance overall, but, like I-TASSER, the average TM-score for subsequences representing 50% or more of their native sequence was similar to that for native sequences, with averages of 0.37 and 0.38, respectively. ProtinfoCM/HH had comparable performance to I-TASSER on native sequences, but lower performance on subsequences. The average TM-score was 0.45 for subsequences representing 50% or more of their native sequence and 0.58 for native sequences. Subsequence length was positively correlated with the prediction quality for subsequences representing 50% or more of the source native sequence (Figure 1). None of the methods could reliably predict structures from subsequences representing less than 50% of the source native sequence, as the average TM-score for these subsequences was less than 0.4 for all three methods.

**Figure 2:**
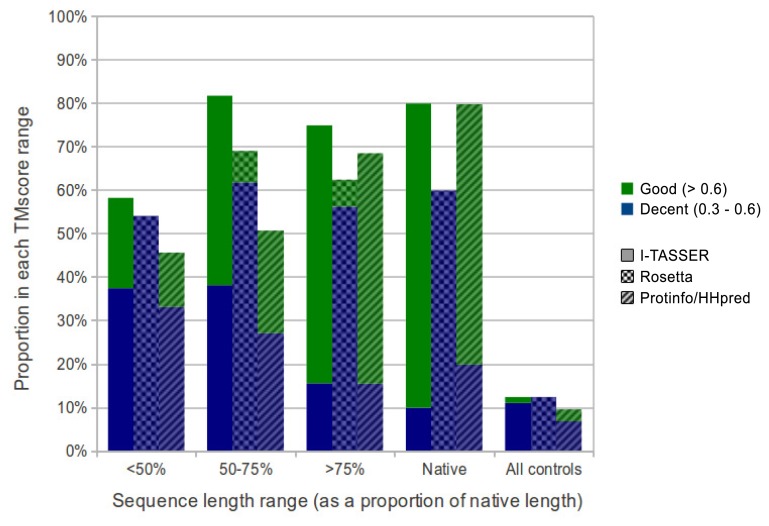
Comparison of the performance of I-TASSER, Rosetta and ProtinfoCM/HH on the native, control and subsequences grouped by sequence length (as a proportion of the length of the source native sequence). Each prediction is defined as “good” (TM-score greater than 0.6), “decent” (TM-score between 0.3 and 0.6) or “bad” (TM-score less than 0.3). The proportion of “bad” predictions within each group are hidden, so that relative method performance is clear. I-TASSER performed best on subsequences of all lengths and native sequences and ProinfoCM/HH performed better than Rosetta on native and longer subsequences, but worse on shorter subsequences, reflecting the bias inherent in the methodologies used (*i.e*., reflecting their capability to infer distant homology).

Overall, I-TASSER performed best on subsequences and native sequences (Figure 2). ProtinfoCM/HH was the second-best performing method on native sequences, as well as the second-best performer on subsequences. Rosetta showed the poorest performance for native sequences and subsequences. MaxSub was also used to assess the quality of each predicted model, and those scores reflected the results discussed above.

### 2.2. Evaluation of Foldability Prediction

**Figure 3:**
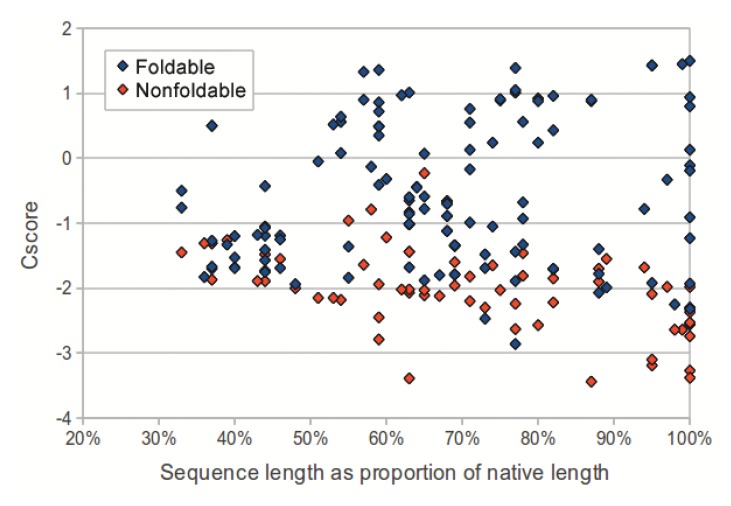
C-score plotted against the subsequence length, measured as a percentage of the length of the source native sequence, for all foldable (blue) and non-foldable (red) sequences. The points at the rightmost side of the plot (100%) represent the native sequences. C-score is a measurement output by I-TASSER that estimates the quality of a predicted model based on the quality of threading alignments and the degree of convergence of structural assembly model refinement simulations. The C-score used for each sequence was the maximum of the five C-scores output for each of the models generated from the input sequence. C-score can effectively distinguish sequences taken from native proteins (foldable) and shuffled versions of those sequences (non-foldable) with accuracy proportional to subsequence length, which has utility in the design of structure prediction and protein design algorithms.

We investigated the utility of using C-score to classify sequences in the dataset as foldable or non-foldable. C-scores were extracted from the I-TASSER models of each sequence in the dataset. The models produced from native sequences and subsequences (labeled as foldable) had higher C-scores than their corresponding shuffled sequences (labeled as non-foldable) (*p*-value: 2.53 × 10^−18^, two-tailed unpaired *t*-test). As sequence length (measured as a proportion of the source native sequence) increases, the C-scores from non-foldable sequences show a greater divergence from those of the foldable sequences (Figure 3). For subsequences that represented less than 50% of the native sequence, the C-scores from the foldable sequences were only marginally higher than those from non-foldable sequences (*p*-value: 0.0321, two-tailed unpaired *t*-test).

**Figure 4:**
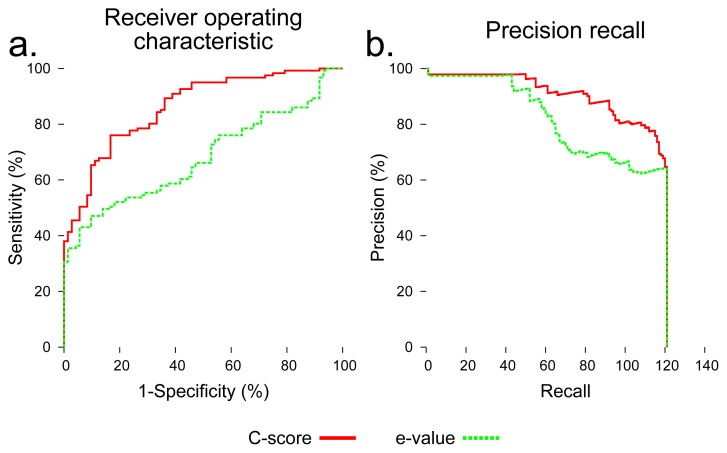
Performance of C-score and BLAST Expect value (E-value) for classifying sequences from the dataset as foldable or non-foldable. Native sequences and subsequences were labeled as foldable, and shuffled versions of those sequences were labeled as non-foldable. C-score is a measurement output by I-TASSER that estimates the quality of a predicted model based on the quality of threading alignments and the degree of convergence of structural assembly model refinement simulations. The C-score used for each sequence was the maximum of the five C-scores output for each of the models generated from the input sequence. BLAST assigns an E-value to each hit resulting from a sequence similarity search that represents the likelihood that the given hit would occur by chance. The E-value used for each sequence is that of the first hit showing 30% or less maximum sequence identity resulting from a search against the NCBI nr database. This value, therefore, reflects how similar the query sequence is to the best-matching hit in the nr database. The prediction accuracy resulting from using each raw score for classification along the range of prediction thresholds was assessed using the following metrics, each of which assess the quality of a binary classifier: (**a**) receiver operating characteristic (ROC) and (**b**) precision recall. Both metrics show that C-score dramatically outperforms E-value, but that both scores show potential utility in predicting foldability.

To evaluate the utility of using C-score to distinguish foldable and non-foldable sequences, we compared BLAST Expect value (E-value) [35] and C-score as predictors of foldability across all sequences in the dataset. C-score dramatically outperformed BLAST E-value in distinguishing foldable and non-foldable sequences (Figure 4).

## 3. Discussion

### 3.1. Structure Prediction of Partial-Length Sequences

The accuracy of I-TASSER and ProtinfoCM/HH relies upon their ability to select templates or template fragments that are homologous to the input sequences. I-TASSER begins the structure prediction process by performing threading to identify template PDB structures that are homologous to the input sequence. Structure fragments excised from the resulting templates are then used to build models. ProtinfoCM/HH is a simpler template-based method that builds a model based on a single template found to be most homologous to the input sequence. Overall, Rosetta performed considerably worse than the other two methods, which was expected given that it is an *ab initio* method. Although Rosetta generates models using template fragments found to be homologous to the input sequence, the fragments it uses to generate models are considerably smaller than the fragments I-TASSER uses to build its models. Unlike the other two methods, on average, Rosetta performed better on subsequences representing more than 50% of the source native sequence than on native sequences. This is likely due to the nature of *ab initio* modeling, which, unlike template-based methods, involves building thousands of models and, then, clustering them and selecting an output model from one of the largest clusters. In *ab initio* modeling, the longer the input sequence, the more models must be generated to produce sizable clusters that contain accurate models, which may explain why predictions made from subsequences were more accurate than those made from the longer native sequences. In our experiment, we generated 10,000 models for each input sequence, a typical number used for structure prediction. If more models are generated, the clustering is more likely to yield a cluster containing an accurate prediction. Therefore, if many more models were generated for each input sequence, the prediction quality of subsequences and native sequences would likely converge.

There were seven control sequences modeled by I-TASSER and three modeled by ProtinfoCM/HH that produced TM-scores greater than 0.4. In all ten cases, the shuffled sequences originated from 2Q2F, a linear alpha helix membrane protein. To investigate the phenomenon, we reshuffled the 2Q2F sequence and found that output structures often showed high TM-scores when aligned to 2Q2F. We hypothesize this is an artifact of TM-score in that it is not unlikely for such a simple linear structure to produce a relatively high TM-score when compared to an arbitrary structure derived from known proteins.

The fact, that all the methods evaluated involve selecting templates that are homologous to the input sequence may explain why the subsequences representing the smallest fractions of the native sequences tended to be the lowest-quality predictions. The smaller the fraction of the native sequence an input subsequence represents, the more difficult it is for the sequence similarity searches used by the structure prediction methods to identify sequences that are homologous to the native sequence. This hypothesis is further supported by the result that in most cases, if the quality of the prediction made from the source native sequence was poor, the subsequence predictions were also poor. This suggests that the reason structures predicted from subsequences are accurate is because threading and sequence similarity searches used in the structure prediction process are able to identify templates homologous to the native sequence, despite having only a partial-length input sequence. Another way to interpret the apparent decline in prediction accuracy as the subsequences get shorter is that a subsequence of a protein *in vitro* or in its biological context may fold differently than the native sequence. The apparent decline in performance given shorter subsequences (Figures 1 and 2) may be the result of accurate predictions of the folded subsequences.

### 3.2. Application of Protein Structure Prediction to EST Data

We have shown that I-TASSER, Rosetta and ProtinfoCM/HHpred can predict the structures of subsequences representing 50% or more of a native protein sequence with accuracy similar to that of structures predicted from native protein sequences. Given that EST sequencing techniques utilize nebulization to randomly fractionate the cDNA before sequencing [36], our benchmarking set of subsequences simulates translated EST sequences. Therefore, if a method, such as ESTScan, is used to predict the protein coding region from high quality EST sequences, and the resulting coding region contains 50% or more of the corresponding native protein sequence, these structure prediction methods can reliably predict the partial protein structure. Additionally, C-scores output by I-TASSER could help to identify ESTs that do not represent real protein sequences. A low C-score for a model produced from a translated EST suggests it would be unlikely to fold into a stable structure. Following structure prediction, an EST structure could be used as a source of information for annotating the EST. The partial structures could be input into automated structure-based function prediction methods, or the structure could be visually inspected by a structure biologist. Functional information derived from accurate EST structures could supplement or validate EST annotations made using existing techniques that rely on sequence information alone. We examined applying structure-based functional analysis tools to the models generated from subsequences, but we were only able to find functional annotations for three out of the ten benchmarking PDB structures. We determined that this sample size would be insufficient to rigorously investigate if subsequence models could be useful for automated functional annotation, but future work should address this question.

### 3.3. Measuring the Foldability of Arbitrary Polypeptide Sequences

The only existing method we are aware of that assigns a single score representing the foldability of an arbitrary amino acid sequence is FoldIndex. FoldIndex was evaluated on a dataset, where the sequences labeled as foldable were full-length native protein sequences and the non-foldable sequences were proteins known to be intrinsically disordered [9]. While these proteins may be a reasonable choice as a set of non-foldable sequences, evaluation of foldability prediction on this dataset alone is not enough to conclude if a method will be reliable for use on arbitrary protein sequences. Intrinsically disordered proteins are relatively rare and poorly-defined; therefore, considering these sequences as a gold standard for arbitrary non-foldable sequences is problematic. We suggest that our dataset of native sequences and subsequences and non-foldable sequences generated by shuffling the residues of the foldable sequences is more reliable and comprehensive for evaluating foldability prediction of arbitrary polypeptide sequences. Experimental evidence has shown that shuffled sequences are very unlikely to produce stable tertiary structures *in vitro* [11]. Shuffling, rather than randomly generating sequences, yields sequences with amino acid compositions that are identical to those of the native sequences. This ensures that any predictive ability observed is not the result of detecting deviations from the amino acid compositions of naturally-occurring proteins.

### 3.4. Foldability Prediction Using C-score

We investigated using C-score as a means of predicting foldability, which we define as the likelihood that a given sequence of amino acids represents a portion of a stable protein structure. Our results demonstrate that C-score can effectively distinguish shuffled and unshuffled sequences from our dataset, which suggests it would be useful for predicting the foldability of arbitrary polypeptide sequences. For comparison, we also evaluated E-value output by a standard BLAST search in the same way C-score was evaluated. E-value represents a naive approach for predicting foldability by making the reasonable assumption that non-foldable sequences would be unlikely to show sequence similarity to known proteins. Although we show that E-value has some predictive power, C-score is much more effective for classifying sequences labeled as foldable or non-foldable. C-score is an estimation of the quality of a structure prediction output by I-TASSER. The score is calculated by measuring the quality alignments produced by matching the query sequence to regions of template structures from the PDB and the degree of structural convergence of assembly refinement simulations. Refinement simulations assemble structure conformations in parallel by combining template fragments and *ab initio* modeling of regions of the query sequence that did not align well to structure fragments from the PDB [4]. Therefore, C-score may be particularly useful for distinguishing foldable and non-foldable sequences, because it is based on both the likelihood that the query sequence is homologous to known structures at the subsequence level, rather than a globally, as performed by BLAST. Furthermore, the score is also derived from structural convergence resulting from *ab initio* modeling, meaning it exploits information from what are essentially constrained simulations of protein folding.

E-value and C-score, however, were not effective for distinguishing shuffled and unshuffled subsequences that represented less than 50% of their source native sequence. Despite that in our experiment, we considered all subsequences derived from native sequences to be foldable, it may be the case that many subsequences representing relatively small portions of native proteins would not in fact fold into stable conformations. Therefore, C-score may have classified the shorter subsequences in our dataset correctly as non-foldable sequences. To better assess the accuracy of using C-score to predict foldability would require evaluation on a dataset where sequences labeled as non-foldable are verified experimentally.

## 4. Experimental Section

### 4.1. Benchmarking Dataset

To generate the benchmarking dataset, a search was performed on the Protein Data Bank (PDB) [37] to select all protein structures that were between 50 and 100 residues in length, had only one chain, were no more than 30% identical to each other by sequence and had an X-ray resolution less than 2.5 Å. The length of each benchmarking protein was limited to 100 residues, because Rosetta does not produce reliable predictions for sequences containing more than 100 residues. Given the 358 structures resulting from the search, TM-score [38] and ClustalW [39] were used to find a subset of ten structures where no structure in the set showed more than 20% sequence similarity or a TM-score greater than 0.35 with respect to the other structures in the set. To simulate ESTs, subsequences were taken from the native sequences corresponding to each of the ten structures in the dataset. A total of 111 subsequences between 40 and 95 residues in length were generated by selecting subsequences beginning at various positions across each native sequence. The length of each subsequence corresponded to a random number generated between 40 and 95. Control sequences were generated for subsequences and native sequences by shuffling the residues of each sequence. In cases where multiple subsequences from a given structure were of the same length, only one control sequence was generated for those subsequences.

### 4.2. Structure Prediction and Model Quality Assessment

All 193 sequences were given as input to the three protein structure prediction methods that were available to be downloaded for local execution. Rosetta3.2 [5] was downloaded from the RosettaCommons website and, for each input sequence, the *ab initio* modeling application was used to generate 10,000 models with the settings recommended in the Rosetta3.2 User Guide for optimal performance. An iterative density protocol [40] was used to select the five final models from the 1000 models with the lowest energy scores given by the Rosetta3.2 scoring function. This approach was found to produce the most accurate final models compared to the others we evaluated. Version 1.1 of the I-TASSER Standalone Package was downloaded from the Zhang Lab website and used without modification on the benchmarking setting to generate models for each input sequence. A modified version of the Protinfo comparative modeling application [6] that omitted the loop building step and selected templates using HHsearch [7] (ProtinfoCM/HH) were also applied to the benchmarking dataset. Templates used by I-TASSER and ProtinfoCM/HH that were more than 30% identical to the input sequence were omitted, which was the default benchmarking setting for I-TASSER. To assess the quality of predictions, TM-score and MaxSub [41] were applied to the final model output by each prediction method. For the methods that output five final models, the score from the best-scoring model was used.

### 4.3. Evaluation of Foldability Prediction

We performed an evaluation using C-score to distinguish native sequences and subsequences (labeled as foldable) from sequences created by shuffling those sequences (labeled as non-foldable). I-TASSER outputs five models for each input sequence, and each of those models is assigned a C-score. The maximum of the five C-scores output for each input sequence was used as a foldability predictor. For comparison, the Expect value (E-value) output by a standard BLAST search was also used as a predictor of foldability. All sequences were searched against the NCBI nr database using the blastp algorithm [35]. The E-value used for each sequence was that of the first hit showing 30% or less maximum sequence identity to the query. All sequences in the benchmarking dataset were assigned a C-score and an E-value, and these values were normalized, so that each score fell between zero and one. These raw scores were then evaluated for their ability to classify each sequence in the dataset as foldable or non-foldable. To assess their predictive utility, a receiver operator characteristic (ROC) curve was generated for C-score and E-value, which plots the balance of specificity and sensitivity across a range of the possible score thresholds between zero and one. Precision recall curves were also generated, which is another assessment technique that compares the precision, the rate of true positives out of all positive predictions, and recall, the proportion of all true positives that were classified correctly.

## 5. Conclusions

We have demonstrated that protein structure prediction software, such as I-TASSER, can be applied to protein sequences representing a fraction of a full-length sequence to reliably predict the partial structure of the source protein. This novel finding suggests that protein structure prediction could be used to improve annotation of ESTs that do not represent full-length protein sequences by confirming that the EST represents a real protein and providing structural information to assist in discovering the function of the EST. Further work should be performed to evaluate if using predicted EST structures does in fact increase the accuracy of EST annotation compared to existing sequence-based methods. Successful application of structure prediction to partial-length sequences also led us to investigate using C-score, an estimation of the quality of an I-TASSER model, to predict the foldability of arbitrary polypeptide sequences. We demonstrated that C-score is highly effective for distinguishing full-length and partial-length sequences from shuffled versions of those sequences. Based on the assumption that shuffled sequences are non-foldable, C-score may represent a useful tool for predicting the foldability of arbitrary polypeptide sequences. Additional evaluation of C-score as a foldability metric using a dataset of experimentally verified foldable and non-foldable sequences would add confidence to our findings.

## Supplementary Information
