# Supplementary material for: Structure Prediction of Partial-Length Protein Sequences
Source: Int J Mol Sci. 2013 Jul 17;14(7):14892–907. doi: 10.3390/ijms140714892 (PMC3742278; doi:10.3390/ijms140714892)
Supplement: Supplementary file 1 [file ijms-14-14892-s001.pdf]

Supplemental data - table of I-TASSER results for all native and subsequences

	Source PDB	Subsequence position	Sequence length (amino acids)	Sequence length (proportion of native)	Native TMscore	Native MaxSub	Control TMscore	Control MaxSub
1	1ABA	1-55	55	0.63	0.5869	0.5783	0.2832	0.2782
2	1ABA	9-63	55	0.63	0.6165	0.6387		
3	1ABA	17-71	55	0.63	0.6303	0.6826		
4	1ABA	1-32	32	0.37	0.4585	0.5617	0.3725	0.4409
5	1ABA	9-40	32	0.37	0.3928	0.4346		
6	1ABA	17-48	32	0.37	0.3188	0.3825		
7	1ABA	25-59	35	0.4	0.3895	0.4296	0.1441	0.1944
8	1ABA	33-67	35	0.4	0.4412	0.5696		
9	1ABA	41-75	35	0.4	0.4078	0.4617		
10	1ABA	25-79	55	0.63	0.5973	0.6511	0.1602	0.1496
11	1ABA	49-82	34	0.39	0.2181	0.3086	0.1706	0.2102
12	1ABA	49-79	31	0.36	0.2595	0.3538	0.1474	0.1958
13	1ABA	Native	87	1	0.7351	0.6991	0.1794	0.142
14	1G6X	1-56	56	0.97	0.6821	0.7233	0.2014	0.1619
15	1G6X	1-45	45	0.78	0.5418	0.6525	0.171	0.1846
16	1G6X	9-53	45	0.78	0.6274	0.7128		
17	1G6X	1-41	41	0.71	0.5654	0.6431	0.1448	0.1706
18	1G6X	11-51	41	0.71	0.5728	0.693		
19	1G6X	1-43	43	0.74	0.5657	0.6485	0.1615	0.1748
20	1G6X	9-51	43	0.74	0.5396	0.6751		
21	1G6X	Native	58	1	0.6394	0.678	0.1736	0.1453
22	1H34	1-47	47	0.82	0.2292	0.2352	0.1557	0.1561
23	1H34	9-55	47	0.82	0.1753	0.1846		
24	1H34	1-51	51	0.89	0.1681	0.1679	0.1705	0.1694
25	1H34	1-56	56	0.98	0.1798	0.1559	0.1467	0.1288
26	1H34	1-50	50	0.88	0.2251	0.2333	0.1549	0.1424
27	1H34	Native	57	1	0.2026	0.1815	0.1598	0.1425
28	1M1Q	1-69	69	0.77	0.3945	0.3582	0.2005	0.1419
29	1M1Q	9-77	69	0.77	0.3112	0.2756		
30	1M1Q	17-85	69	0.77	0.2679	0.2251		
31	1M1Q	1-66	66	0.73	0.4014	0.3908	0.1949	0.1732
32	1M1Q	9-74	66	0.73	0.3521	0.33		
33	1M1Q	17-82	66	0.73	0.2309	0.2044		
34	1M1Q	25-65	41	0.46	0.1793	0.2049	0.1545	0.1732
35	1M1Q	33-73	41	0.46	0.1603	0.1954		
36	1M1Q	41-81	41	0.46	0.2093	0.2672		
37	1M1Q	25-64	40	0.44	0.2195	0.2641	0.192	0.2395
38	1M1Q	33-72	40	0.44	0.1617	0.1962		
39	1M1Q	41-80	40	0.44	0.1992	0.2723		
40	1M1Q	49-87	39	0.43	0.1727	0.1963	0.1854	0.2369
41	1M1Q	49-88	40	0.44	0.1874	0.2188	0.1419	0.1677
42	1M1Q	Native	90	1	0.1717	0.1196	0.2076	0.1065
43	1NEG	1-49	49	0.75	0.9063	0.9553	0.163	0.1728
44	1NEG	9-57	49	0.75	0.8677	0.9339		
45	1NEG	1-53	53	0.82	0.8831	0.9333	0.1656	0.1434
46	1NEG	9-61	53	0.82	0.8386	0.8877		
47	1NEG	25-65	41	0.63	0.6686	0.7244	0.1687	0.1864
48	1NEG	25-65	41	0.63	0.6284	0.7118	0.1684	0.1926
49	1NEG	Native	65	1	0.843	0.8473	0.1795	0.1392
50	2BK8	1-57	57	0.59	0.5821	0.6257	0.2348	0.2191

51	2BK8	11-67	57	0.59	0.7995	0.8535		
52	2BK8	21-77	57	0.59	0.8564	0.9127		
53	2BK8	1-84	84	0.87	0.8781	0.8644	0.211	0.1207
54	2BK8	11-94	84	0.87	0.8687	0.8681		
55	2BK8	21-72	52	0.54	0.7526	0.8397	0.1548	0.1175
56	2BK8	31-82	52	0.54	0.8172	0.9038		
57	2BK8	41-92	52	0.54	0.8037	0.895		
58	2BK8	21-77	57	0.59	0.8548	0.9112	0.1428	0.1041
59	2BK8	31-87	57	0.59	0.8731	0.9255		
60	2BK8	41-89	49	0.51	0.8465	0.9274	0.1617	0.1223
61	2BK8	41-91	51	0.53	0.7858	0.8869	0.1656	0.1309
62	2BK8	Native	97	1	0.8357	0.8079	0.2245	0.112
63	2NSN	1-76	76	0.8	0.9058	0.9184	0.2373	0.195
64	2NSN	9-84	76	0.8	0.8404	0.8516		
65	2NSN	17-92	76	0.8	0.7784	0.7817		
66	2NSN	1-67	67	0.71	0.8927	0.9176	0.2516	0.2202
67	2NSN	9-75	67	0.71	0.8874	0.9208		
68	2NSN	17-83	67	0.71	0.8543	0.877		
69	2NSN	25-66	42	0.44	0.723	0.8812	0.2708	0.2929
70	2NSN	33-74	42	0.44	0.6629	0.7837		
71	2NSN	41-82	42	0.44	0.4744	0.524		
72	2NSN	25-86	62	0.65	0.7601	0.7871	0.2259	0.2137
73	2NSN	33-94	62	0.65	0.6223	0.6611		
74	2NSN	49-94	46	0.48	0.4608	0.5023	0.2029	0.1963
75	2NSN	49-79	31	0.33	0.5872	0.8335	0.2679	0.3367
76	2NSN	57-87	31	0.33	0.6663	0.8466		
77	2NSN	Native	95	1	0.7552	0.7365	0.2405	0.122
78	2Q2F	1-49	49	0.68	0.584	0.5836	0.4869	0.4759
79	2Q2F	9-57	49	0.68	0.5682	0.5768		
80	2Q2F	17-65	49	0.68	0.4708	0.4974		
81	2Q2F	1-56	56	0.78	0.6303	0.6242	0.441	0.479
82	2Q2F	9-64	56	0.78	0.43	0.4541		
83	2Q2F	1-49	49	0.68	0.5863	0.5847	0.4848	0.475
84	2Q2F	9-57	49	0.68	0.5716	0.578		
85	2Q2F	17-65	49	0.68	0.4719	0.4967		
86	2Q2F	1-68	68	0.94	0.6209	0.6116	0.5921	0.5564
87	2Q2F	25-67	43	0.6	0.2336	0.2311	0.4347	0.4396
88	2Q2F	25-66	42	0.58	0.2188	0.2185	0.3901	0.4152
89	2Q2F	25-71	47	0.65	0.3945	0.4236	0.4343	0.466
90	2Q2F	25-70	46	0.64	0.4353	0.4612	0.2419	0.2332
91	2Q2F	Native	72	1	0.6965	0.6879	0.6323	0.6359
92	3HGL	1-54	54	0.69	0.3662	0.3528	0.2778	0.2772
93	3HGL	9-62	54	0.69	0.2093	0.1926		
94	3HGL	17-70	54	0.69	0.3069	0.3068		
95	3HGL	1-54	54	0.69	0.3653	0.3523	0.2032	0.209
96	3HGL	9-62	54	0.69	0.2093	0.1948		
97	3HGL	17-70	54	0.69	0.307	0.307		
98	3HGL	1-69	69	0.88	0.3031	0.2547	0.2278	0.2165
99	3HGL	9-77	69	0.88	0.1737	0.1412		
100	3HGL	1-74	74	0.95	0.2911	0.2576	0.2335	0.1471
101	3HGL	25-67	43	0.55	0.1525	0.1817	0.1957	0.2048
102	3HGL	33-75	43	0.55	0.1142	0.1144		

103	3HGL	25-76	52	0.67	0.2177	0.2301	0.1979	0.213
104	3HGL	25-73	49	0.63	0.2154	0.2186	0.1787	0.1798
105	3HGL	25-75	51	0.65	0.2309	0.2401	0.1533	0.1522
106	3HGL	Native	78	1	0.3949	0.3444	0.2188	0.154
107	3LLB	1-80	80	0.99	0.9085	0.9158	0.1704	0.1015
108	3LLB	1-77	77	0.95	0.8773	0.89	0.1723	0.1238
109	3LLB	1-77	77	0.95	0.8741	0.8891	0.1924	0.1079
110	3LLB	1-62	62	0.77	0.8356	0.8812	0.1788	0.1337
111	3LLB	9-70	62	0.77	0.8278	0.8761		
112	3LLB	17-78	62	0.77	0.8582	0.899		
113	3LLB	25-70	46	0.57	0.739	0.8398	0.2199	0.2434
114	3LLB	33-78	46	0.57	0.8439	0.9261		
115	3LLB	25-72	48	0.59	0.7276	0.8086	0.1784	0.1688
116	3LLB	33-80	48	0.59	0.846	0.9204		
117	3LLB	25-74	50	0.62	0.7478	0.8055	0.1847	0.1734
118	3LLB	25-75	51	0.63	0.7463	0.801	0.1929	0.1585
119	3LLB	49-78	30	0.37	0.7113	0.9369	0.1265	0.181
120	3LLB	49-78	30	0.37	0.7099	0.9361	0.1264	0.1811
121	3LLB	Native	81	1	0.8853	0.8945	0.1898	0.1237
